# Cancer classification: Mutual information, target network and strategies of therapy

**DOI:** 10.1186/2043-9113-2-16

**Published:** 2012-10-02

**Authors:** Wen-Chin Hsu, Chan-Cheng Liu, Fu Chang, Su-Shing Chen

**Affiliations:** 1 System Biology Lab, University of Florida, Florida, USA; 2 Department of Electrical and Computer Engineering, University of Florida, Florida, USA; 3 Department of Computer and Information Science and Engineering, University of Florida, Florida, USA; 4 Institute of Information Science, Academia Sinica, Taipei, Taiwan

**Keywords:** Feature selection, Biomarkers, Microarray, Therapy design, Target network

## Abstract

**Background:**

Cancer therapy is a challenging research area because side effects often occur in chemo and radiation therapy. We intend to study a multi-targets and multi-components design that will provide synergistic results to improve efficiency of cancer therapy.

**Methods:**

We have developed a general methodology, AMFES (Adaptive Multiple FEature Selection), for ranking and selecting important cancer biomarkers based on SVM (Support Vector Machine) classification. In particular, we exemplify this method by three datasets: a prostate cancer (three stages), a breast cancer (four subtypes), and another prostate cancer (normal vs. cancerous). Moreover, we have computed the target networks of these biomarkers as the signatures of the cancers with additional information (mutual information between biomarkers of the network). Then, we proposed a robust framework for synergistic therapy design approach which includes varies existing mechanisms.

**Results:**

These methodologies were applied to three GEO datasets: GSE18655 (three prostate stages), GSE19536 (4 subtypes breast cancers) and GSE21036 (prostate cancer cells and normal cells) shown in. We selected 96 biomarkers for first prostate cancer dataset (three prostate stages), 72 for breast cancer (luminal A vs. luminal B), 68 for breast cancer (basal-like vs. normal-like), and 22 for another prostate cancer (cancerous vs. normal. In addition, we obtained statistically significant results of mutual information, which demonstrate that the dependencies among these biomarkers can be positive or negative.

**Conclusions:**

We proposed an efficient feature ranking and selection scheme, AMFES, to select an important subset from a large number of features for any cancer dataset. Thus, we obtained the signatures of these cancers by building their target networks. Finally, we proposed a robust framework of synergistic therapy for cancer patients. Our framework is not only supported by real GEO datasets but also aim to a multi-targets/multi-components drug design tool, which improves the traditional single target/single component analysis methods. This framework builds a computational foundation which can provide a clear classification of cancers and lead to an efficient cancer therapy.

## Background

Cancer therapy is a difficult research area due to its level of complexity. Lately, the mere superposition of single drugs is found to generate side-effects and crosstalk with another drug which may cancel out the final success of treatments. Thus, current research focuses on measuring the drug treatments as a whole rather than considering them individually [1,2]. Later, a synergistic concept is proposed to evaluate the drug treatments [3]. However, evaluations are still based on cases and do not have a systematic approach. In [4], a network methodology is first used to evaluate efficiency of drug treatments. Thus, Li et al. use a parameter, namely a SS (Synergy Score) to introduce the topology factor of the network based on the disease and the drug agent combination [5].

Our approach is first to build a more precise target network from the selected biomarkers (by AMFES) [6]. Then, we identify the intrinsic properties by computing mutual information of the interactions among these biomarkers. Our approach is to improve Li’s results by considering the mutual information in the target network. And we provide a general framework of synergistic therapy, which may include several different approaches.

## Methods

### AMFES

The COD (Curse of Dimensionality) has been a major challenge of microarray data analysis due to the large number of genes (features) and relatively small number of samples (patterns). To tackle this problem, many gene selection methodologies were developed to select only significant subsets of genes in a microarray dataset. AMFES selects an optimal subset of genes by training a SVM with subsets of genes generated adaptively [6].

When AMFES runs a dataset, all samples are randomly divided into a training subset *S* of samples and a testing subset *T* of samples at a heuristic ratio of 5:1. *S* is used for ranking and selecting of genes and for constructing a classifier out of the selected genes. *T* is used for computing test accuracy. When a training subset *S* is given, we extract *r* training-validation pairs from *S* according to the heuristic rule *r* = max (5, (int) (500/*n*+0.5)) and *n* is the number of samples in *S*. Each pair randomly divides *S* into a training component of samples and a validation component of samples at a ratio of 4:1. The heuristic ratio and rule are chosen based on the experimental experiences at the balance of time consumption and performance. Basically, AMFES has two fundamental processes, ranking and selection. We first explain each process in details and then the integrated version at the end.

### Ranking

The gene ranking process contains a few ranking stages. At first stage, all genes are ranked by their ranking scores in a descending order. Then, in the next stage, only the top half ranked genes are ranked again while the bottom half holds the current order in the subsequent stage. The same iteration repeats recursively until only three genes are remained to be ranked again to complete one ranking process. Assume at a given ranking stage, there are *k* genes indexed from *1* to *k*. To rank these *k* genes, we follow 4 steps below. (I) We first generate *m* independent subsets *S*_*1*_*… S*_*m*._ Each subset *S*_*i*_, *i* = 1, 2… *m*, has *j* genes which are selected randomly and independently from the *k* genes, where *j* = (int) (*k*/2). (II) Let C_*i*_ be the SVM classifier that is trained on each subset of genes_*,*_*i* = 1, 2… *m*. For each gene of *k* genes, we compute the ranking score $\theta_{m}$ (g) of the gene *g*, as equation (1). (III) We use the average weight of the gene *g*, the summation of weights of *g* in *m* subsets divided by the number of subsets for which *g* is randomly selected. This increases the robustness to represent the true classifying ability of the gene *g*. (IV) Rank *k* genes in the descending order by their ranking scores.

(1) $$\theta_{m}\left(g\right)=\frac{\sum_{i=1}^{m}I_{\left\{g\in S_{i}\right\}}weighti\left(g\right)}{\sum_{i=1}^{m}I_{\left\{g\in S_{i}\right\}}}$$

where I is an indicator function such that I_proposition_ = 1 if the proposition is true; otherwise, I_proposition_ = 0. In other word, if gene *g* is randomly selected for the subset *S*_*i*_, it is denoted as $g\in S_{i}$ and I_proposition_ = 1.

We denote the objective function of C_*i*_ as $obj_{i}\left(v_{1},v_{2},\ldots ,v_{s}\right)$ where **v**_1_, **v**_2_… **v**_s_ are support vectors of C_*i*_. The *weight*_*i*_(g) is then defined as the change in the objective function due to g, i.e.,

(2) $$weight_{i}\left(g\right)=\left|obj_{i}\left(v_{1},v_{2},\ldots v_{s}\right)-obj_{i}\left(v_{1}^{\left(g\right)},v_{2}^{\left(g\right)},\ldots ,v_{3}^{\left(g\right)}\right)\right|$$

[6][7,8]. Note that if **v** is a vector, **v**^(*g*)^ is the vector obtained by dropping gene *g* from **v**. Let θ_m_ be a vector comprising the ranking scores derived from the *m* gene subsets generated thus far and θ_m-1_ is the vector at the previous stage. The *m* value is determined when θ_m_ satisfies the equation (3) by adding a gene to an empty subset once a time.

(3) $$\frac{{\left\|\theta_{m-1}\;-\;\theta_{m}\right\|}^{2}}{{\left\|\theta_{m-1}\right\|}^{2}}<\;0.01$$

where ||θ|| is understood as the Euclidean norm of vector θ. The pseudo codes of ranking process are shown in below.

#### *Pseudo codes for ranking process of AMFES*

RANK-SUBROUTINE

INPUT: a subset of k genes to be ranked

Generate k artificial genes and put them next to the original genes

Pick an initial tentative value of m

DO WHILE m does not satisfies equation (3)

FOR each subset Si of m subsets

Randomly select j elements from k genes to form the subset Si.

Train an SVM to get weight_*i*_(g) for each gene in the subset

ENDFOR

FOR each gene of k genes

Compute the average score of the gene from m subsets

ENDFOR

List k genes in descending order by their ranking scores

ENDDO

OUPUT: a ranked k genes

### Selection

Ranking artificial features together with original features has been demonstrated as a useful tool to distinguish relevant features from irrelevant ones as in [9-11]. In our selection process, we also use this technique to find the optimal subset of genes.

Assume a set of genes is given. We generate artificial genes and rank them together with original ones. After finishing ranking the set, we assign a gene-index to each original gene by the proportion of artificial ones that are ranked above it where the gene-index is the real numerical value between 0 and 1. Then, we generate a few subset candidates from which the optimal subset is chosen. Let *p*_*1*_, *p*_*2*_, be the sequence of subset-indices of the candidates with *p*_*1*_ < *p*_*2*_ < ….where *p*_*i*_ = *i*×0.005 and *i*= 1,2,…200. Let B(*p*_*i*_) denote the corresponding subset of subset-index *p*_*i*_*,* and it contains original genes whose indices are smaller than or equal to *p*_*i*_*.* Then*,* we train a SVM on every B(*p*_*i*_), and compute its validation accuracy *v*(*p*_*i*_).

We stop at the first *p*_*k*_ at which v(*p*_*k*_) ≥ *v*_*baseline*_ and *v*(*p*_*k*_) ≥ *v*(*p*_*l*_) for *k* ≤ *l* ≤ *k*+10, where *v*_*baseline*_ is the validation accuracy rate of the SVM trained on the baseline, i.e., the case in which all features are involved in training. The final result, B(*p*_*k*_), is then the optimal subset for the given set of genes. The pseudo codes for selection process of AMFES are listed below.

#### *Pseudo codes for selection process of AMFES*

SELECTION-SUBROUTINE

INPUT: a few subsets with their validation accuracies, av(p _*i*_ )

Compute the validation accuracy of all genes, vbaseline.

FOR each subset given

IF v(p _*k*_) ≥ v_*baseline*_ and v(p_*k*_) ≥ v(p_*l*_) for k ≤ l ≤ k+10 THEN

*Resulted subset is B(p*_*k*_*)*

ENDIF

ENDFOR

*OUPUT: B(p*_*k*_*)*

### Integrated version

The ranking and selection processes from previous sections are for one training- validation pair. To increase the reliability of validation, we generate *r* pairs to find the optimal subset. We calculate the validation accuracy of the *q*^th^ pair for all *p*_*q-i*_ subsets where *q* denotes pair-index and *i* denotes the subset-index. Then, we compute *av*(*p*_*i*_), the average of *v*(*p*_*q-i*_) over *r* training-validation pairs and perform the subset search as explained in selection section on *av*(*p*_*i*_) to find the optimal *p*_*i*_, denoted as p*.However, p* does not correspond to a unique subset, since each pair has its own B(p*) and they can be all different. Thus, we adopt all samples of *S* as training samples in order to find a unique subset. We generate artificial genes and rank them together with original genes. Finally, we select the original genes whose indices are smaller than or equal to the p* as the genes we select for *S*. The integrated version of process is shown below. In the pseudo codes below, the AMFES-ALGORITHM represents the integrated version of the whole process while RANK-SUBROUTINE represents the ranking process and SELECTION-SUBROUTINE represents the selection process.

#### *Pseudo codes for integrated version of AMFES*

AMFES ALGORITHM-Integrated Version

INPUT: a dataset

*Divide a dataset into train samples and testsamples.*

Divide the train samples into r training-validation components pairs

FOR each pair of r train-validation components pairs

*Generate 200 candidate subsets p*_*q-*__*i*_

FOR each subset of 200 subsets

CALL RANK subroutine to rank each subset.

Assign each original gene a gene-index

Train each subset on an SVM and compute corresponding validation accuracy, v(p_*q-i*_), for the subset

END FOR

END FOR

FOR each subset of 200 subsets

Compute average validation rate, av(p_*i*_), of the subsetfrom r pairs.

END FOR

CALL SELECTION subroutine to search for the optimal subset by its average validation rate and denotes it as p*

CALL RANK subroutine to rank original genes again and select original genes which belong to the subset B(p*).

OUPUT: an optimal subset of genes B(p*)

### Mutual information

Mutual information has been used to measure the dependency between two random variables based on the probability of them. If two random variables X and Y, the mutual information of X and Y, I(X; Y), can be expressed as these equivalent equations [12]:

(4) $$I\left(X;Y\right)=H\left(X\right)-H\left(X|Y\right)$$

(5) $$=H\left(Y\right)-H\left(Y|X\right)$$

(6) $$=H\left(X\right)+H\left(Y\right)-H\left(X,Y\right)$$

where H(X), H(Y) denote marginal entropies, H(X|Y) and H(Y|X) denote conditional entropies and H(X,Y) denotes joint entropy of the X and Y. To compute entropy, the probability distribution functions of the random variables are required to be calculated first. Because gene expressions are usually continuous numbers, we used the kernel estimation to calculate the probability distribution [13].

Assume the two random variables X and Y are continuous numbers. The mutual information is defined as [12]:

(7) $$I\left(X,Y\right)=\int \int f\left(x,y\right)\log\left(\frac{f\left(x,y\right)}{f\left(x\right)f\left(y\right)}\right)dxdy$$

where *f*(x,y) denotes the joint probability distribution, and *f*(x) and *f*(y) denote marginal probability distribution of X and Y. By using the Gaussian kernel estimation, the *f*(x, y),*f*(x) and *f*(y) can be further represented as equations below [14]:

(8) $$f\left(x,y\right)=\frac{1}{M}{\sum_{2\pi h^{2}}e}^{-\;\frac{1}{2h^{2}}\left({\left(x-x_{u}\right)}^{2}+\left(y-y_{u}^{2}\right)\right)}$$

(9) $$f\left(x\right)=\frac{1}{M}\Sigma \frac{1}{\sqrt{2\pi h^{2}}}e^{-\frac{1}{2h^{2}}{\left(x-y_{u}\right)}^{2}}\text{,}$$

where *M* represents the number of samples for both X and Y, *u* is index of samples u=1,2,…M, and *h* is a parameter controlling the width of the kernels. Thus, the mutual information $I\left(X,Y\right)$ can then be represented as:

(10) $$I\left(X,Y\right)=\frac{1}{M}\sum_{i}\log\frac{M{\sum_{i}e}^{-\frac{1}{2h^{2}}\left({\left(x_{w}-x_{u}\right)}^{2}+{\left(y_{\mathrm{wi}}-y_{u}\right)}^{2}\right)}}{\sum_{j}e^{-\frac{1}{2h^{2}}{\left(x_{w}-x_{u}\right)}^{2}}\sum_{j}e^{-\frac{1}{2h^{2}}{\left(y_{\mathrm{wi}}-y_{u}\right)}^{2}}}$$

where both *w, u* are indices of samples w,u=1,2,…M.

Computation of pairwise genes of a microarray dataset usually involves nested loops calculation which takes a dramatic amount of time. Assume a dataset has *N* genes and each gene has *M* samples. To calculate the pairwise mutual information values, the computation usually first finds the kernel distance between any two samples for a given gene. Then, the same process goes through every pair of genes in the dataset. In order to be computation efficient, two improvements are applied [13]. The first one is to calculate the marginal probability of each gene in advance and use it repeatedly during the process [13][15].The second improvement is to move the summation of each sample pair for a given gene to the most outer for-loop rather than inside a nested for-loop for every pairwise gene. As a result, the kernel distance between two samples is only calculated twice instead *N* times which saves a lot of computation time. LNO (Loops Nest Optimization) which changes the order of nested loops is a common time-saving technique in computer science field [16].

### Target network

The effect of drugs with multiple components should be viewed as a whole rather than a superposition of individual components [1][2]. Thus, a synergic concept is formed and considered as an efficient manner to design a drug [3]. In [17], mathematical models are used to measure the effect generated by the multiple components. However, it does not consider practical situation such as crosstalk between pathways. A network approach starts to be used to analyze the interactions among multiple components [4]. Initiated by work in [4], another system biological methodology, NIMS (Network-target-based Identification of Multicomponent Synergy) is proposed to measure the effect of drug agent pairs depending on their gene expression data [5]. NIMS focuses on ranking the drug agent pairs of Chinese Medicine components by their SS.

In [5], it assumes that a drug component is denoted as a drug agent and with which a set of genes associated are denoted as agent genes of the drug agent. For a given disease, assume there are *N* drug agents where *N* =1, 2…*n*. Initially, NIMS randomly chooses two drug agents from *N*, A_1_, and A_2_, and builds a background target network by their agent genes in a graph. From the graph, NIMS calculates TS (Topology Score) of the graph by applying the PCA (Principle Component Analysis) to form a IP value which is integrated by betweenness, closeness and a variant of Eigenvalues PageRank [18]. The TS is used to evaluate the topology significance of the target network for the drug agent pair, A_1_ and A_2_, and is defined as

(11) $$TS_{1,2}=\frac{1}{2}\times \left[\frac{\sum_{i}IP_{1}\left(i\right)\times \exp\left(-\min\left(d_{i,j}\right)\right)}{\sum_{i}IP_{1}\left(i\right)}+\frac{\sum_{j}IP_{2}\left(j\right)\times \exp\left(-\min\left(d_{j,i}\right)\right)}{\sum_{j}IP_{2}\left(j\right)}\right]\text{,}$$

where *IP*_1_ and *IP*_2_ denote IP values for drug agent A_1_ agent and A_2_. Min(*d*_i,j_) denotes minimum shortest path from gene *i* of A_1_ to all genes of A_2_ and min(*d*_j,i_) denotes the one from gene *j* of A_1_ to all genes of A_2_.

NIMS define another term, AS (Agent Score), to evaluate the similarity of a disease phenotype for a drug agent. For a given drug agent, if one of its agent genes has a phenotype record in the OMIM (Online Mendelian Inheritance in Man) database, the drug agent has that phenotype as one of its phenotype. The similarity score of a drug agent pair is defined as the cosine value of the pair’s feature vector angle [19]. The AS is defined as:

(12) $$AS_{1,2}=\frac{\underset{i,j}{\sum }P_{i,j}}{M},$$

where *P*_i,j_ denotes similarity score of *i*th phenotype of A_1_ and *j*th phenotype of A_2_ and *M* denotes the total number of phenotypes.

The SS of the pair is then defined as the product of TS and AS. NIMS calculates SS for all possible drug agent pairs for a disease and then can find potential drug agent pairs after ranking them by SS.

## Results

### MIROARRAY data description

We made a brief description of these three datasets in Table 1. It listed the number of biomarkers, types of biomarkers, number of samples and variation of samples used.

**Table 1:** **Descriptions of 3 datasets:GSE18655 (prostate cancer), GSE19536(breast cancer) and GSE21036(prostate cancer)**

	**Prostate Cancer (GSE18655)**	**Breast Cancer (GSE19536)**	**Prostate Cancer (GSE21036)**
Number of Biomarkers	502	489	373
Type of Biomarkers	RNAs	miRNAs	miRNAs
Number of Samples	139	78	142
Variation of Samples	Grade1(38), Grade2(90), Grade3(11)	Luminal A ( 41), Luminal B (12), Basal-like (15), Normal-like(10)	Cancerous (114), Normal(28)

#### The prostate cancer dataset with RNA biomarkers

In order to give a better prognosis, pathologists have used a cancer stage to measure cell tissues and tumors’ aggressions as an indicator for doctors to choose a suitable treatment. The most widely used cancer staging system is TNM (Tumor, Node, and Metastasis) system [20]. Depending on levels of differentiation between normal and tumor cells, a different histologic grade is given. Tumors with grade 1 indicate almost normal tissues, with grade 2 indicating somewhat normal tissues and with grade 3 indicating tissues far away from normal conditions. Although most of cancers can be adapted to TNM grading system, some specific cancers require additional grading systems for pathologists to better interpret tumors.

The Gleason Grading System is especially used for prostate cancers and a GS (Gleason Score) is given based on cellular contents and tissues of cancer biopsies from patients. The higher the GS are, the worse the prognoses are. The prostate cancer dataset, GSE18655, includes 139 patients with 502 molecular markers, RNAs [21]. In [21], it showed that prostate tumors with gene fusions, TMPRSS2: ERG T1/E,4 have higher risk of recurrences than tumors without the gene fusions. 139 samples were prostate fresh-frozen tumor tissues of patients after a radical prostatectomy surgery. All samples were taken from the patients’ prostates at the time of prostatectomy and liquid nitrogen was used to freeze middle sections of prostates at extreme low temperature. Among these patients, 38 patient samples have GS 5–6 corresponding to histologic grade 1, 90 samples have GS 7 corresponding to histologic grade 2 and 11 samples have GS 8–9 corresponding to histologic grade 3. The platform used for the datasets is GPL5858, DASL (cDNA-mediated, annealing, selection, extension and ligation) Human Cancer Panel by Gene manufactured by Illumina. The FDR (false discovery rate) of all RNAs expressions in the microarray is less than 5%.

#### Breast cancer dataset with Non-coding miRNA biomarkers

The miRNAs have strong correlation with some cellular processes, such as proliferation, which has been used as a breast cancer dataset [22]. It has 799 miRNAs and 101 patients’ samples. Differential expressions of miRNAs indicated different level of proliferations corresponding to 6 intrinsic breast cancer subtypes: luminal A, luminal B, basal-like, normal-like, and ERBB2. The original dataset has 101 samples and among them, 41 samples are luminal A, 15 samples are basal-like, 10 samples are normal-like, 12 samples are luminal B, 17 samples are ERBB2, 2 samples have T35 mutation status, another sample has T35 wide type mutation and 3 samples are not classified. GSE19536 was represented in two platforms GPL8227, an Agilient-09118 Human miRNA microarray 2.0 G4470B (miRNA ID version) and the GPL6480, an Agilent-014850 whole Human Genome Microarray 4x44k G4112F (Probe Name). For this paper, we only used the expressions from GPL8227.

#### Prostate cancer dataset of cancerous and normal samples with miRNA biomarkers

The CNAs (Copy Number Alterations) of some genes may associate with growth of prostate cancers [23]. In addition, some changes are discovered in mutations of fusion gene, mRNA expressions and pathways in a majority of primary prostate samples. The analysis was applied to four platforms and consists of 3 subseries, GSE21034, GSE21035 and GSE21036 [23]. For this paper, we only use the GSE 21036 for analysis. The microarray dataset has 142 samples which include 114 primary prostate cancer samples and 28 normal cells samples. The platform is Agilent-019118 Human miRNA Microarray 2.0 G4470B (miRNA ID version).

### Results of AMFES

We employ the AMFES on the prostate cancer (GSE18655), breast cancer (GSE19536) and another prostate cancer (GSE21036) datasets. Consequently, for GSE18655, AMFES selects 96 biomarkers. The classification is performed in two steps. The first step performs classification between grade1 and above samples and it selects 93 biomarkers. At the second step, AMFES classifies between grade2 and grade3 samples and it selects 3 biomarkers. Thus, we can assume that these 96 biomarkers can classify among grade1, grade2 and grade3 samples [6]. For GSE19536, AMFES also performs classification in two steps. At the first step, AMFES classify between luminal and non-luminal types samples and it selects 47 biomarkers [6]. At the second step, AMFES further classifies luminal samples into luminal A and luminal B and selects 27 biomarkers. For the non-luminal samples, AMFES also classifies them into basal-like and normal-like samples and selects 25 biomarkers [6]. After removing duplicate biomarkers, AMFES has 72 (47+27-2(duplicated)) for classifying luminal samples and 68 (47+25-4(duplicated)) for classifying non-luminal ones [6]. For GSE21036, AMFES simply selects 22 biomarkers for classifying cancerous and normal samples. Table 2. shows the number of selected genes. The complete lists of these biomarkers can be found in Additional file 1 GSE18655_96_Biomarkers.xlsx, Additional file 2 GSE19536_72_Biomarkers.xlsx, Additional file 3 GSE19536_68_Biomarkers.xlsx, and Additional file 4 GSE21036_22_Biomakers.xlsx.

**Table 2:** **Results of selected subsetsof genes**

	**Prostate Cancer (GSE18655)**	**Breast Cancer (GSE19536)**	**Breast Cancer (GSE19536)**	**Prostate Cancer (GSE21036)**
Number of Biomarkers Selected	96	72	68	22
Variation of Samples	Grade1, Grade2, Grade3	Luminal A, Luminal B	Basal-like Normal-like	Cancerous Normal

We then apply the MI calculation described in the Mutual Information section on 96 biomarkers for GSE18655 and represent the pairwise MI values of grade 1, grade 2 and grade 3 samples in three 96*96 matrixes which can be found in Additional file 5 GSE18655 Grade1 MI.xlsx, Additional file 6 GSE18655 Grade2 MI.xlsx and Additional file 7 GSE18655 Grade3 MI.xlsx. We also represent the four MI matrixes of 72 and 68 biomarkers for GSE19536 in Additional file 8 GSE19536 Luminal-A MI.xlsx, Additional file 9 GSE19536 Luminal-B MI.xlsx, Additional file 10 GSE19536 Basal-Like MI.xlsx, and Additional file 11 GSE19536 Normal-Like MI.xlsx. The two MI matrixes for GSE21036 are in Additional file 12 GSE21036 Cancer MI.xlsx, Additional file 13 GSE21036 Normal MI.xlsx.

**Figure 1:**
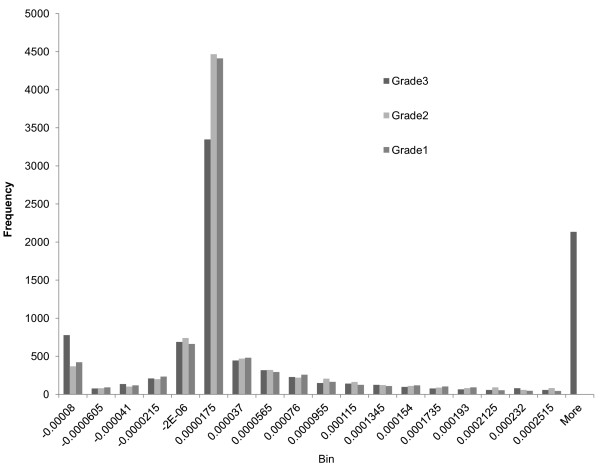
Comparison of 96 MI of grade1, grade2 and grade3 samples.

**Figure 2:**
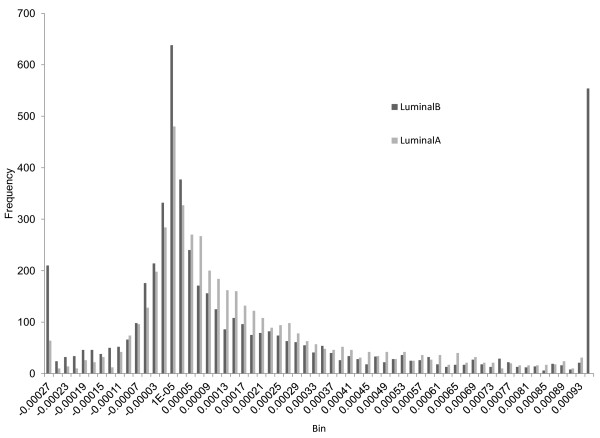
Comparison of 72 MI of luminal A and luminal B samples.

**Figure 3:**
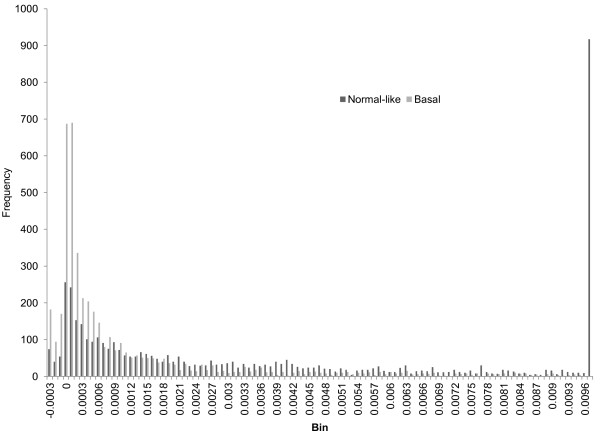
Comparison of 68 MI of basal-like and normal-like samples.

**Figure 4:**
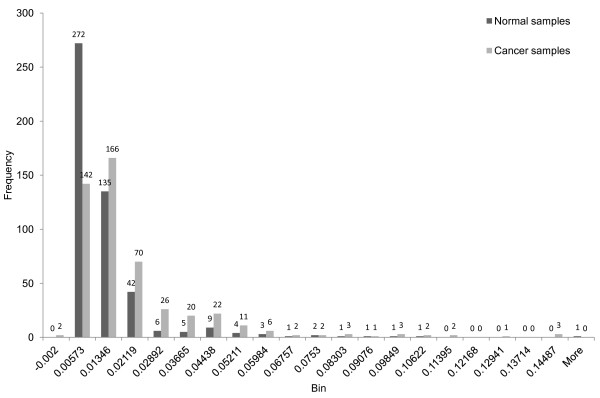
Comparison of 22 MI of prostate cancerous and normal samples.

We analyze these MI matrixes and list differences between them under different conditions in Table 3. For a given matrix, the first column in Table 3 denotes the mean value; the second column denotes the standard deviation; the third column shows the number of positive values in the matrix; the fourth column shows the number of negative values; the sixth column shows the minimum value and the seventh column displays the maximum. In the fifth column, we compare MI matrixes under two different conditions such as luminal A vs. luminal B. If the signs of two entries at the same position in these two matrixes are different, we count it as one sign difference. The fifth column denotes the number of sign differences of the samples compared. We employ the same process for comparing basal-like versus normal-like for GSE19536 and the cancerous versus normal for GSE21036. To visualize the differences, we display the histograms of MI values of grade1s, grade2s and grade3s in Figure 1. Figure 2 shows the histograms for luminal As versus luminal Bs. Figure 3 shows basal-likes versus normal-likes and Figure 4 shows the cancerous versus normals.

**Table 3:** **Results of analysis ofMI matrices**

	**Mean value of MI**	**Standard deviation of MI**	**Num of positive values**	**Num of negative values**	**Num of values ofdifferent sign**	**Min value**	**Max value**
GSE18655_grade1	0.00024	0.0015	6298	2918	N/A	−0.0011	0.0858
GSE18655_grade2	0.00020	0.0017	6468	2748		−0.0018	0.0949
GSE18655_grade3	0.0004	0.0021	6650	2566		−0.0029	0.0582
GSE19536_A(72)	0.00036	0.0022	3912	1272	2052	−0.0010	0.1293
GSE19536_B(72)	0.00053	0.0040	3388	1796		−0.0022	0.2279
GSE19536_BasalLike(68)	0.0017	0.0056	3491	998	1217	−0.0033	0.1648
GSE19536_NormalLike(68)	0.0056	0.008	4200	420		−0.002	0.1279
GSE21036_cancer	0.0165	0.0212	10	474	56	−0.002	0.1446
GSE21036_norm	0.0086	0.0146	46	438		−0.0015	0.1565

For the fifth column of comparison of GSE18655, since there are three types prostate, they cannot be fairly compared, so we skipped the process for it. In addition, because there are many MI entries for all histograms, we only show the densest section of each histogram in figures.

### Results of calculating mutual information

The statistic results of calculating mutual information are shown in Table 3 at the end of this paper.

### Synergistic therapy

Based on the interpretation of the network [4,5], we proposed a framework that can help to elucidate the underlying interactions between multi-target biomarkers and multi-component drug agents. The framework consists of three parts: selecting biomarkers of a complex disease such as cancer, building target networks of biomarkers, and forming interaction between biomarkers and drug agents to provide a personalized and synergistic therapy plan.

From the GEO datasets of cancers, we have discovered the genetic model of each cancer, called signature of that particular cancer. Among different cancers, their signatures (target networks) may be quite different which corresponds to different biomarkers in Additional file 1 GSE18655_96_Biomarkers.xlsx, Additional file 2 GSE19536_72_Biomarkers.xlsx, Additional file 3 GSE19536_68_Biomarkers.xlsx, and Additional file 4 GSE21036_22_Biomakers.xlsx. For these different signatures, we would discover various synergistic mechanisms which have exemplified in [24].

**Figure 5:**
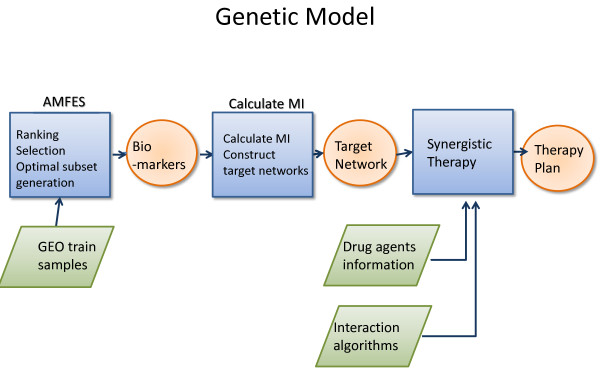
Diagram of detailed process of building the genetic model.

Assume we would like to provide a synergistic therapy plan of a patient A. By collecting his/her bodily data such as saliva, blood samples, we first obtain the corresponding microarray dataset of patient A and apply it to the genetic model as shown in Figure 5.

**Figure 6:**
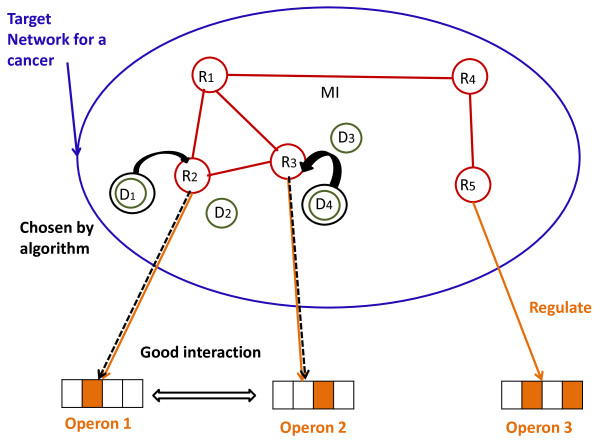
Relationships between biomarkers, pharmacons and operons where R_1_, R_2_, R_3_, R_4_ and R_5_ denote 5 biomarkers. Among all the biomarkers, R_2_, R_3_ and R_5_ are regulators.

A complete synergistic therapy should be able to select small subset of biomarkers and correlate them with drug agents in a multi-target multi-components network approach as shown in Figure 6. In Figure 6, a disease associates with several biomarkers such as RNAs, miRNAs or proteins denoted by R_1_, R_2_, R_3_, R_4_ and R_5_ which are the regulators for operons O_1_, O_2_, and O_3_. An operon is a basic unit of DNAs and formed by a group of genes controlled by a gene regulator. These operons initiate molecular mechanisms as promoters. The gene regulators can enable organs to regulate other genes either by induction or repression. For each target biomarker, it may have a list of pharmacons used as enzyme inhibitors. Traditionally, pharmacons are referred to biological active substances which are not limited to drug agents only. For example, the herbal extractions whose ingredients have a promising anti-AD (Alzheimer’s Disease) effect can be used as pharmacons [24]. Meanwhile, pharmacons denoted by D_1_, D_2_, and D_3,_ have effects for some target biomarkers. For example, D_1_ affects target biomarker R_3_, D_2_ affects target biomarker R_5_ and D_3_ affects biomarker R_1_. Compared with drug agent pair methodology [5], the proposed framework in Figure 6 represents a more accurate interpretation of biomarkers with multi-component drug agents.

## Discussion

Among the MI values obtained, we see positive values and negative values. The positive value can represent the attractions among the biomarkers while the negative may represent the repulsion among the biomarkers, which matches the concept of Yin-Yang in TCM (Traditional Chinese Medicine). From these results, we observed that there is minimal difference of mutual information values between cancer stages. However, the difference of mean MI value of the prostate cancer versus normal cells is move obvious. The mean MI value of the last prostate cancer cell is approximately twice that of normal cells. This may be intriguing for medical people for further investigations.

## Conclusions

We have presented a comprehensive approach to diagnosis and therapy of complex diseases, such as cancer. A complete procedure is proposed for clinical application to cancer patients. While the genetic model provides a standard framework to design synergistic therapy, the actual plan for individual patient is personalized and flexible. With careful monitoring, physicians may adaptively change or modify the therapy plan. Much further analysis of this framework in clinical settings should be experimented.

## Competing interests

The authors declare that they have no competing interests.

## Author’s contributions

WH, CL: Implementation of project. FC, SC: Design the project. All authors read and approved the final manuscript.

## Supplementary Material

Additional file 1 **GSE18655_96_Biomarkers.** An MS Office Excel file which contains a list of gene symbols of 96 biomarkers of GSE18655 samples. Click here for file

Additional file 2 **GSE19536_72_Biomarkers.** An MS Office Excel file which contains a list of gene symbols of 72 biomarkers of GSE19536 luminal A and luminal B samples. Click here for file

Additional file 3 **GSE19536_68_Biomarkers.** An MS Office Excel file which contains a list of gene symbols of 68 biomarkers of GSE19536 basal-like and normal-like samples. Click here for file

Additional file 4 **GSE21036_22_Biomarkers.** An MS Office Excel file which contains a list of gene symbols of 22 biomarkers of GSE21036 samples. Click here for file

Additional file 5 **18655 Grade1 MI.** An MS Office Excel file which contains a matrix of the pairwise MI values of 96 biomarkers of grade1 samples. Click here for file

Additional file 6 **18655 Grade2 MI.** An MS Office Excel file which contains a matrix of the pairwise MI values of 96 biomarkers of grade2 samples. Click here for file

Additional file 7 **18655 Grade3 MI.** An MS Office Excel file which contains a matrix of the pairwise MI values of 96 biomarkers of grade3 samples. Click here for file

Additional file 8 **19536 Luminal-A MI.** An MS Office Excel file which contains the pairwise MI values of 72 biomarkers of luminal A samples. Click here for file

Additional file 9 **19536 Luminal-B MI.** An MS Office Excel file which contains the pairwise MI values of 72 biomarkers of luminal B samples. Click here for file

Additional file 10 **19536 Basal-Like MI.** An MS Office Excel file which contains the pairwise MI values of 68 biomarkers of Basal-like samples. Click here for file

Additional file 11 **19536 Normal-Like MI.** An MS Office Excel file which contains the pairwise MI values of 68 biomarkers of Normal-like samples. Click here for file

Additional file 12 **21036 Cancer MI.** An MS Office Excel file which contains the pairwise MI values of 22 biomarkers of cancerous samples. Click here for file

Additional file 13 **21036 Normal MI.** An MS Office Excel file which contains the pairwise MI values of 22 biomarkers of normal samples. Click here for file
